# Fatty Acid Amide Hydrolase in Prostate Cancer: Association with Disease Severity and Outcome, CB_1_ Receptor Expression and Regulation by IL-4

**DOI:** 10.1371/journal.pone.0012275

**Published:** 2010-08-19

**Authors:** Lina Thors, Anders Bergh, Emma Persson, Peter Hammarsten, Pär Stattin, Lars Egevad, Torvald Granfors, Christopher J. Fowler

**Affiliations:** 1 Pharmacology Unit, Department of Pharmacology and Clinical Neuroscience, Umeå University, Umeå, Sweden; 2 Pathology Unit, Department of Medical Biosciences, Umeå University, Umeå, Sweden; 3 Departments of Oncology and Radiation Sciences, Umeå University, Umeå, Sweden; 4 Urology and Andrology Unit, Department of Surgical and Perioperative Sciences, Umeå University, Umeå, Sweden; 5 Department of Pathology and Cytology, Karolinska University Hospital, Stockholm, Sweden; 6 Department of Urology, Central Hospital, Västerås, Sweden; Bauer Research Foundation, United States of America

## Abstract

**Background:**

Recent data have indicated that there may be a dysregulation of endocannabinoid metabolism in cancer. Here we have investigated the expression of the endocannabinoid metabolising enzyme fatty acid amide hydrolase (FAAH) in a well characterised tissue microarray from patients diagnosed with prostate cancer at transurethral resection for voiding problems.

**Methodology/Principal Findings:**

FAAH immunoreactivity (FAAH-IR) was assessed in formalin-fixed paraffin-embedded non-malignant and tumour cores from 412 patients with prostate cancer. CB_1_ receptor immunoreactivity (CB_1_IR) scores were available for this dataset. FAAH-IR was seen in epithelial cells and blood vessel walls but not in the stroma. Tumour epithelial FAAH-IR was positively correlated with the disease severity at diagnosis (Gleason score, tumour stage, % of the specimen that contained tumour) for cases with mid-range CB_1_IR scores, but not for those with high CB_1_IR scores. For the 281 cases who only received palliative therapy at the end stages of the disease, a high tumour epithelial FAAH-IR was associated with a poor disease-specific survival. Multivariate Cox proportional-hazards regression analyses indicated that FAAH-IR gave additional prognostic information to that provided by CB_1_IR when a midrange, but not a high CB_1_IR cutoff value was used. Interleukin-4 (IL-4) receptor IR was found on tumour epithelial cells and incubation of prostate cancer PC-3 and R3327 AT1 cells with IL-4 increased their FAAH activity.

**Conclusions/Significance:**

Tumour epithelial FAAH-IR is associated with prostate cancer severity and outcome at mid-range, but not high, CB_1_IR scores. The correlation with CB_1_IR in the tumour tissue may be related to a common local dysregulation by a component of the tumour microenvironment.

## Introduction

Prostate cancer is the commonest cancer form in males. According to the U.S. National Cancer Institute (http://www.cancer.gov/cancertopics/types/prostate), the number of new cases of prostate cancer in the United States in 2009 was 192,280, and the number of deaths due to the disease was 27,360. Treatment options range from expectancy to prostatectomy, hormonal treatment and radiation depending upon the nature of the cancer.

In recent years, evidence has accumulated to suggest that the endocannabinoid system, comprising the cannabinoid CB_1_ and CB_2_ receptors, their endogenous ligands anandamide and 2-arachidonoylglycerol, and their synthetic and degradative enzymes, plays a role in the pathogenesis and possible treatment of cancer types, including prostate cancer (for recent reviews, see [1]–[3]). Thus, for example, cannabinoids produce a CB receptor-mediated apoptosis in glioma cells as a result of a sustained production of ceramide [4] and several papers have reported both CB receptor-dependent and -independent effects of cannabinoids upon the viability, mobility and invasivity of both androgen-sensitive and insensitive prostate cancer cell lines [5]–[11].

*In vitro*, a local endocannabinoid tone is an important factor controlling the malignancy of prostate cancer cells. Thus, modulation of the levels of endocannabinoids, by use of inhibitors of endocannabinoid synthesis or metabolism, results in a change in the invasive properties of prostate cancer cells in a manner consistent with a protective effect of endocannabinoids [8], [12]. The endocannabinoid anandamide is metabolised by the enzyme fatty acid amide hydrolase (FAAH) and overexpression of the enzyme in PC-3 cells results in a phenotype with an increased invasive behaviour *in vitro* [12]. Those authors concluded that “inhibition of FAAH activity by specific inhibitors may be one therapeutic target for the treatment of prostate cancer” [12]. FAAH inhibitors, which do not produce the sort of central behavioural effects associated with cannabinoids like Δ^9^-tetrahydrocannabinol [13], are currently in drug development, primarily with pain as a therapeutic target, and the leading compound is in phase II of its development.

Less is known about the endocannabinoid system in prostate tumour tissue, but in a study using a large number of patients with a long follow-up, we recently reported that the expression of CB_1_ receptors in tumour tissue obtained at diagnosis was associated with disease severity and outcome [14]. Thus, individuals with a CB_1_ receptor immunoreactivity (CB_1_IR) above or equal to the median score for the data set had a higher incidence of metastases at diagnosis, and a higher proportion of cases with a high Gleason score (an indicator of disease severity based on the morphology of the two most common tumour types in the sample [15]) than those individuals with a CB_1_IR below the median for the data set. Furthermore, for the individuals who had been followed by expectancy until appearance of metastases, a high CB_1_IR score was associated with a lower disease-specific survival [14]. With respect to FAAH, it has been reported that in a commercially available tissue microarray, cores from prostate cancer tissues had a higher expression of the enzyme than seen in normal tissue [12]. However, no data on the relation between the FAAH immunoreactivity (FAAH-IR) and disease outcome was presented. In addition, possible reasons for the high level of FAAH-IR in the tumour tissue were not investigated by these authors. In consequence, in the present study, we have investigated the FAAH-IR in the tissue microarray used previously by us to characterise CB_1_IR in the tumour tissue [14], and undertaken studies using cultured cells to see if a known component of the tumour microenvironment can affect the FAAH activity. Further, we have investigated whether FAAH-IR is correlated with other pathogenetic and prognostic variables, such as the local tumour stage number and the immunoreactive score for phosphorylated epidermal growth factor receptor (pEGFR).

## Methods

### Ethics Statement

The research ethical committee at Umeå university hospital (Regional Ethical Review Board in Umeå, Sweden) approved of the study and waived the need for informed consent.

### Patients

The formalin-fixed, paraffin-embedded samples used in the present study were collected at the Central Hospital, Västerås, Sweden, between 1975 and 1991 from a total of 412 patients diagnosed with prostate cancer at transurethral resection for micturation difficulties [16]. The presence of metastases was determined using a bone scan shortly after the transurethral resection. The patients were followed until 2003. For the 388 cases where tumour FAAH-IR could be scored (see below), 281 were followed by watchful waiting until the appearance of metastases (the standard treatment approach at the time). The other patients received hormonal treatment, radiotherapy or radical prostatectomy. The Gleason scores and the percentage of the specimen that contained tumour were assessed in each sample. Cause of death was assessed by evaluation of medical records. Tissue microarrays (cores with a diameter of 0.6 mm) were constructed using a Beecher Instrument (Sun Prairie, WI, USA). Each tissue microarray slide (56 in total, usually with cores from 8 cases per slide) contained up to eight (usually five) samples of tumour tissue (both primary and secondary Gleason grade areas were included) and up to four (usually four) samples of non-malignant tissue from each patient [16].

### Immunohistochemistry

Sections were deparaffinized, rehydrated and placed in citrate buffer pH 6.0. After boiling in a pressure cooker for 60 min, samples were placed in water and then in Ventana buffer, after which they were placed in a Ventana automated analyser (Ventana Medical Systems Inc., Tucson, AZ) to which the FAAH antibody (dilution 1/2000) and the secondary system (iVIEW DAB Detection Kit, Ventana Medical Systems Inc.) were added. The antibody used, a rabbit anti-FAAH raised against the last 102 amino acids of rat FAAH, has been characterised in detail [17] and was found to produce the appropriate staining pattern (somatodendritic staining of principal cells) in formalin-fixed tissue from human hippocampus (data not shown), consistent with the rat hippocampus [18], [19]. CB_1_ receptor immunoreactivity (CB_1_IR) scores were available in the database, and have been reported elsewhere [14].

The individual cores were scored under the microscope for FAAH-IR by an investigator (LT) who was blind to the clinical data for the patients. The scoring system used was essentially the same as that used previously for CB_1_IR [14]. In brief, for the epithelial tissue, the cores were scored for immunoreactive intensity (0–3 where 0 is absent and 3 is high) and distribution within the epithelial tissue (0, 10, 25, 33, 50, 67 or 100% of the total distribution for each intensity). The distribution for the most common intensity was set to 100 minus the sum of the other intensities. The fractional distribution at each intensity was multiplied with the intensity score and the values summed to give a final score for the core. Thus, for example, a core with intensities of 1 (10%), 2 (10%) and 3 (remainder) (distribution in brackets) would receive a composite score of 1×0.1+2×0.1+3×0.8 = 2.7. When identifiable in the core, the basal and luminal epithelial tissue was scored separately. Blood vessel walls were scored in the same way, but with a simpler distribution (0, 50 and 100%). Throughout the scoring, examples of cores scored as 1, 2 and 3 were on hand for comparison as an “internal standard”. Cores where the structure or quality of the tissue was not sufficiently good or where the staining was unclear were excluded from the study. For each measure, the median values for the composite scores for each patient were calculated and entered into the database by a second investigator (CF). As a measure of the reliability of the tumour epithelial scores, 67 cores (16 patients) were scored at two separate occasions by the same investigator. The Spearman correlation coefficient for the individual core scores between this pilot and the main study was 0.76 (n = 67, p<0.0001, 95% confidence interval 0.63–0.85). The intraclass correlation coefficient for single measures (ICC(2,1), a measure of the reproducibility of the scoring method) was 0.67 (95% confidence interval 0.51–0.78, p<0.001).

Interleukin-4 (IL-4) receptor immunoreactivity was assessed in formalin fixed, paraffin embedded tissue slices using a monoclonal antibody to the extracellular domain of the recombinant human IL-4 receptor (MAB230, R&D Systems Inc., Minneapolis, USA). The antibody dilution was 1/200 and the immunoreaction was detected using the Ventana detection system as above. Lymph node tissue served as a positive control.

### Culturing of cells

PC-3 and LNCaP human prostate cancer cells (passage ranges 32–38 and 14–19, respectively), and R3327 AT-1 rat prostate cancer cells (passage range 49–59) were available at the Department. PC-3 cells were cultured in Hams F-10, 2mM L-glutamine, 10% foetal bovine serum and 100 ml^−1^ penicillin +100 g ml^−1^ streptomycin (PEST). LNCaP cells were cultured in RPMI 1640, 2 mM Glutamine, 10% foetal bovine serum and PEST. R3327 AT-1 cells were cultured in RPMI 1640, 250 nM dexamethasone, 10% foetal bovine serum and PEST. P19 mouse embryonic carcinoma cells (passage range 17–19) were obtained from the European Collection of Cell Cultures (Porton Down, UK). P19 cells were cultured in MEM alpha 22571 with 10% foetal bovine serum, 1% nonessential amino acids and and PEST. Cells were grown in 75 cm^2^ culturing flasks at 37°C with 5% CO_2_ in humidified atmospheric pressure. Cells were passaged twice a week and cell culture medium was changed three times a week.

### Interleukin-4 (IL-4) stimulation of FAAH activity in cultured cancer cells

The cells were plated at a density of 1×10^6^ cells per well in 6-well plates. Following incubation overnight at 37°C in an atmosphere of 5% CO_2_, the cell culture medium was changed, to medium supplemented with IL-4, and incubated for an additional 24 hours. Cells were washed twice with ice-cold phosphate-buffered saline (PBS) and thereafter collected using a rubber policeman. On ice, cells were resuspended in PBS, centrifuged for 5 minutes at 1000×g and pellet resuspended in 10 mM Tris buffer at pH 9. The homogenates were stored at −80°C in aliquots until used. The protein content was determined using bovine serum albumin as standard [20].

The FAAH activity was measured as described by Boldrup et al. [21]. Briefly, following incubation of homogenates with 2 µM of [^3^H]AEA labelled in the ethanolamine part of the molecule (assay pH 9), a charcoal mixture (80 µl charcoal +300 µl 0.5 M HCl per tube) were added and tubes were placed on ice to stop the reaction. The charcoal was then sedimented by centrifugation, 2500×g for 10 minutes, and aliquots of the supernatant were transferred to scintillation vials and tritium content was determined by liquid scintillation spectroscopy with quench correction. Blank values are defined as the accumulated amount of tritium for assays performed in the absence of homogenates.

### RNA extraction and cDNA synthesis

PC-3 and LNCaP cells were plated at a density of 1×10^6^ cells per well in 6-well plates and treated with IL-4 as described above. For PCR analysis, total RNA was extracted using the miRNeasy Kit (Qiagen, Hilden, Germany) according to the instructions by the manufacturer. The RNA concentration was measured using a Nanodrop instrument (Thermo Scientific, Wilmington, DE, USA), and cDNA was synthesized from 2 µg of total RNA using a cDNA synthesis kit with random primers (High capacity cDNA reverse transcription kit; Applied Biosystems, Foster City, CA, USA). For semi-quantitative PCR, samples from the same treatment group were pooled (n = 6).

### Semi-quantitative PCR

The mRNA levels for human CB_1_, FAAH and GAPDH were analysed using a Taq PCR Core kit (Qiagen, Hilden, Germany), a Biometra TProfessional thermocycler (Biometra, Göttingen, Germany) and the following primers: hFAAH (197 bp), forward 5′-TGGAAGTCCTCCAAAAGCCCAG-3′, reverse 5′- TGTCCATAGACACAGCCCTTCAG-3′, hCB_1_ (165 bp), forward 5′- AAGGTGACATGGCATCCAAAT-3′, reverse 5′-AGGACGAGAGAGACTTGTTGTAA-3′, and hGAPDH (180 bp), forward 5′- CAACTACATGGTTTACATGTTC-3′, reverse 5′- GCCAGTGGACTCCACGAC-3′. The conditions for PCR were: denaturing at 94°C for 2 min, annealing for 40 s at 57°C (GAPDH) or 60°C (FAAH, CB_1_) followed by elongation at 72°C for 60 s; in subsequent cycles denaturing was performed at 94°C for 40 s. For FAAH and CB_1_, the analyses included 35 cycles, whereas 25 cycles were used for GAPDH.

### Quantitative real-time PCR

In addition to semi-quantitative PCR on pooled samples, analysis of individual samples was performed using quantitative real-time PCR. Both SYBR Green (FAAH, CB_1_, GAPDH) and TaqMan (ß-actin) kinetics were used. For FAAH, CB_1_ and GAPDH, the primers described above were used together with Power SYBR Green PCR Master Mix (Applied Biosystems, Foster City, CA, USA), whereas human ß-actin was detected using a pre-made primer-probe mix (Hs00357333_g1, Applied Biosystems) together with TaqMan Universal PCR Master Mix (Applied Biosystems). The amplifications were performed on an ABI PRISM 7900 HT Sequence Detection System and software (Applied Biosystems), and the samples were analysed in triplicates. Both GAPDH and ß-actin were used as housekeeping genes, with similar results (data for ß-actin not shown). The relative expression of FAAH and CB_1_was calculated from *Ct* values using the standard curve method.

### Western Blot

For Western blotting, protein samples were electrophoresed on 10% SDS – polyacrylamide gels under reducing conditions and fractionated proteins were electrophoretically transferred onto Immobilon-P membrane (Millipore, Bedford, MA, USA). Membranes were blocked over night at 4°C on an orbital shaker in 5% non-fat powdered milk, 0.05% Tween-20 in PBS (PBS- T) prior to incubation with primary rabbit polyclonal antibody against FAAH (1∶1000–1∶5000; the same antibody as used in the immunohistochemical part of the study) or primary rabbit polyclonal antibody against actin (1∶8000; Sigma-Aldrich, St Louis, MO, USA). Primary antibodies were diluted in PBS-T 3% (FAAH) or PBS-T 5% (actin) non-fat powdered milk. After incubation with peroxidase-conjugated secondary antibodies (Amersham Biosciences), proteins were detected using enhanced chemiluminescence detection system (Amersham Biosciences). Molecular sizes of protein bands were determined by parallel electrophoresis of molecular weight markers (Bio-Rad Laboratories AB, Sundbyberg, Sweden). Protein concentrations were determined by BSA protein assay kit (Pierce, Rockford, IL, USA).

### Compounds

Anandamide (ethanolamine-1-^3^H; specific activity: 2.2 TBqmmol^−1^) was obtained from American Radiolabeled Chemicals Inc. (St Louis, MO, USA). Unlabelled anandamide and URB597 were obtained from the Cayman Chemical Co. (Ann Arbor, MI, USA). Interleukin-4 was obtained from Sigma-Aldrich.

### Statistics

With the exception of the Cox proportional-hazards regression and intraclass correlation coefficient analyses, which were conducted using SPSS software (SPSS Inc., Chicago, IL, USA), all statistical calculations were undertaken using the statistical package built into the GraphPad Prism 5 computer programme for the Macintosh (GraphPad Software Inc., San Diego, CA, USA). For survival analyses, an event was defined as death due to prostate cancer and entered into the database as “event = 1”. Death from other causes was censored, as were the cases where the patient was still alive at the date of last follow-up. These were grouped as “event = 0”. Cases (n = 3) where the disease outcome was unknown were excluded from the survival analyses. The duration of event-free survival is defined as the time from diagnosis until the date of prostate cancer death, death of other causes, or if no death occurred, until the date of last follow-up.

Receiver operating characteristic (ROC) curves were used to define cut-offs for FAAH-IR, which were then used to construct survival curves using the Kaplan-Meier method. Differences in outcome between groups were tested with the log-rank test. Cox proportional-hazards regression analyses were undertaken to assess the influence of the FAAH score upon the outcome at different CB_1_IR cutoffs. Differences in the distribution of variables between groups of patients were analyzed using the chi-square test.

For readers unfamiliar with the statistical methods used to assess the influence of FAAH-IR upon disease outcome, a brief explanation of their usefulness is warranted, particularly with respect to the choice of cutoff values for FAAH-IR. The ROC test was originally developed to aid interpretation of radar signals, and plots the number of true negatives (1- the false positive fraction, termed 1 - specificity) *vs.* true positives (sensitivity) at a given cutoff value (i.e. for cases with a score equal to, or higher than, the selected value). The area under the ROC curve can then be calculated. For a test with absolutely no diagnostic value, the curve will be a straight line with an area of 0.5. A perfect test would give a value of 1.0, so the further away from 0.5 the value, the better the test. For a test with an area under the curve significantly greater than 0.5, an important issue is the choice of cutoff, the value chosen as a signal that the patient may have the disease in question. If the cutoff value is set low, then the sensitivity will be high, but there will be a large number of false positives. If the cutoff value is set high, then the number of false positives will be lower, but a large number of true positives will be missed. The choice of cutoff is thus a trade-off between maximising the number of true positives found and minimising the number of false positives. Choice of cutoff is dependent upon the relative importance of missing true positives *vs.* misdiagnosing true negatives for the disease in question, but a useful way of identifying an optimal cutoff is to determine the cutoff point with the maximal vertical distance between the observed value and the corresponding value for a test with no diagnostic value, calculated as maximum sensitivity + specificity -1. This value is termed the Youden index, J, and we have used this value here for FAAH-IR (termed J_FAAH_). For useful reviews on ROC curves and the choice of optimal cutoff values, see [22]–[24].

With respect to the statistical analyses of survival curves, the Kaplan-Meier method, where differences in outcome between groups is tested with the log-rank test, provides a useful (and visual) method for assessing differences in survival curves. The Cox proportional-hazards regression analyses the hazard “as a function of the explanatory variables and unknown regression coefficients multiplied by an arbitrary and unknown function of time” (citation from the original paper of Cox [25]), i.e. assesses the contribution of different potential risk factors on the end-point measured (in this case, death due to prostate cancer) without making assumptions about the basic survival curve itself.

## Results

### Distribution of FAAH immunoreactivity in non-malignant and tumour tissue

FAAH-IR was scored in cores from a total of 412 patients. Of these, records for six patients were not found in the data base, and so data for these patients were not included in the analyses.

**Figure 1:**
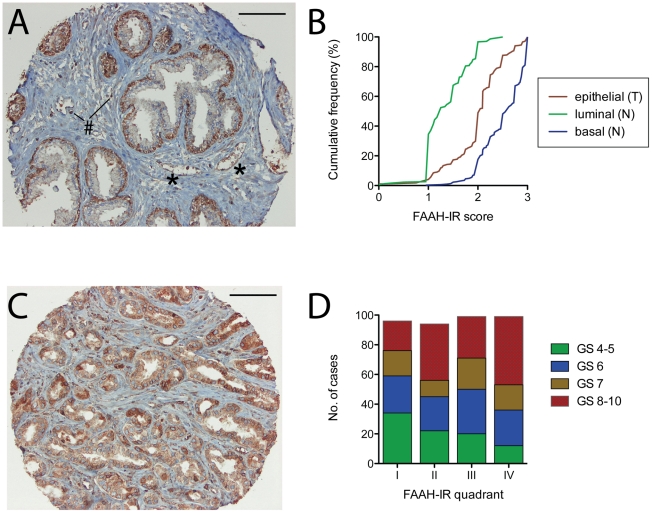
FAAH immunoreactivity in formalin-foxed prostate cancer samples. Panel A. Example of FAAH-IR in a core of non-malignant prostate tissue obtained at transurethral resection from a 75 year old patient. The asterisks indicate blood vessels, and the # symbol indicates FAAH positive cells, presumably infiltrating lymphocytes. In Panel C, the FAAH-IR in a core of tumour tissue from a 64 year old patient is shown. The lines at the top right above the cores indicate 100 µm. Panel B shows the cumulative frequencies of FAAH-IR scores for the basal and luminal non-malignant (N) epithelial tissue (n = 307 in both cases) and for the tumour (T) epithelial tissue (n = 388). The median values (with interquartile range in brackets) were: basal (N), 2.625 (2.125–2.9); luminal (N), 1.25 (1–1.75); epithelial (T), 2.05 (1.8–2.3). The median values were significantly different from each other (p<0.001, Dunn's multiple comparison test following significant Kruskal-Wallis test). Panel D shows the relation of the epithelial tumour FAAH-IR to the Gleason score (GS) at diagnosis. The FAAH-IR scores were divided into quadrants where I refers to scores between <1.8 (n = 96), II between 1.8 and 2 (n = 94), III between 2.05 and 2.25 (n = 99) and IV between 2.3 and 3 (n = 99). The distribution of the GS groups was significantly different in the quadrants (χ^2^ = 27.03, df = 9, p<0.0005).

An example of the FAAH-IR for the non-malignant tissue is shown in Fig. 1A. FAAH-IR was seen in the epithelial cells, and in blood vessels, as well as in cells (possibly lymphocytes, which express FAAH [26]) infiltrating the stroma in some cores. In all, 1247 cores from 377 patients were scored for non-malignant epithelial FAAH. Of these, 733 cores from 307 cases had definable basal : luminal morphology and the FAAH-IR for these cases were scored separately and used in the analyses. In addition, 993 cores from 369 cases were scored for FAAH-IR in the blood vessel walls. The cumulative distribution of the scores in the basal and luminal epithelial cells is shown in Fig. 1B, where it is clear that the median FAAH-IR is higher in the basal epithelial cells than in the luminal cells. In fact, of the 733 cores scored, the basal FAAH-IR score was higher than the corresponding luminal score in 724 cores and the two scores were equal in 9 cores. The luminal score was never higher than the basal score. The blood vessel FAAH-IR was scored slightly differently, so a direct comparison of the values is not relevant. However, there was a significant correlation between basal FAAH-IR and blood vessel FAAH-IR, but not between the luminal and blood vessel FAAH-IR (Table 1).

**Table 1:** Correlation matrix for FAAH-IR in tumour and non-malignant tissue.

	Spearman's r [no of pairs in correlation] for FAAH-IR in:				
	Tumour samples (T)		Non-malignant tissue samples (N)		
parameter	epithelial	blood vessel	basal	luminal	blood vessel
Epithelial FAAH (T)	-	0.37***[357]			
Epithelial FAAH (N):					
basal			-	0.54***[307]	0.21***[299]
luminal				-	0.08^NS^[299]
Epithelial CB_1_IR (T)	0.23***[370]	0.13*[344]			
Epithelial CB_1_IR (N)			0.11∧[293]	0.04^NS^[293]	0.04^NS^[336]

***p<0.001; *p<0.05, ∧not significant (p<0.06), ^NS^not significant (p>0.16). CB_1_IR values are taken from the original data of [14]. The scores for the epithelial FAAH (T), basal (but not luminal) FAAH (N), blood vessel FAAH (T and N), and epithelial CB_1_IR (T and N) were not normally distributed (p<0.0001, D'Agostino & Pearson omnibus normality test).

A total of 1851 cores from 388 cases were scored for FAAH-IR in the tumour epithelial cells. For the blood vessels, 1201 cores from 360 patients were scored. An example of the staining for a tumour core is shown in Fig. 1C and the cumulative distribution of the epithelial scores are shown in Fig. 1B, where it can be seen that the median score was significantly higher than the non-malignant luminal score, but lower than the basal score.

The samples used here have previously been investigated by us with respect the expression of epithelial CB_1_ receptors [14]. For the non-malignant samples, there was no significant correlation between the CB_1_IR scores and either basal, luminal or blood vessel FAAH-IR. In contrast, a significant correlation between the tumour CB_1_IR and the tumour epithelial and blood vessel FAAH-IR was seen (Table 1).

### FAAH activity in prostate cancer cell lines is affected by interleukin-4

**Figure 2:**
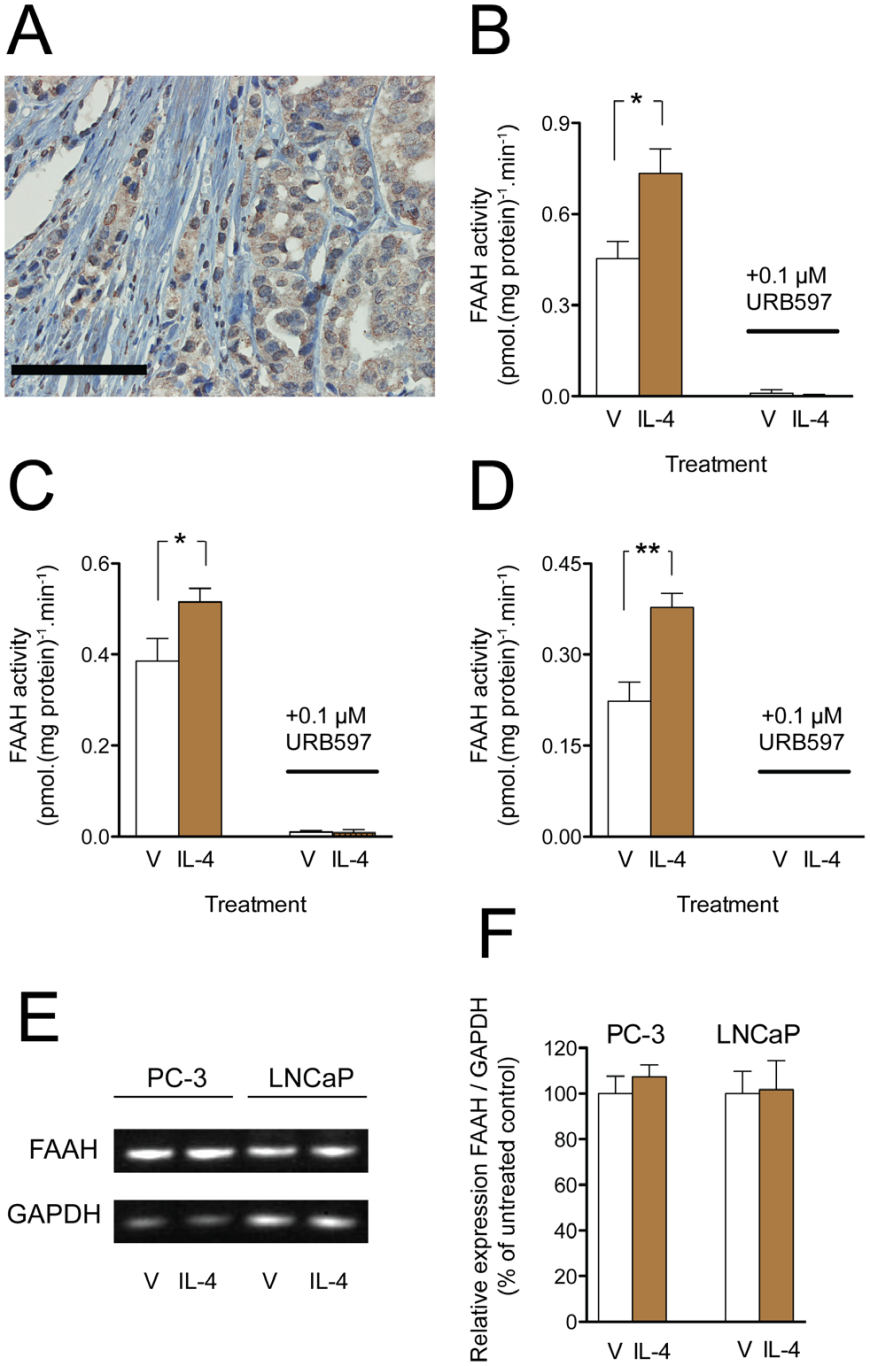
IL-4 affects FAAH activity in prostate cancer cells. Panel A, IL-4 receptor immunoreactivity in a prostate tumour tissue sample (Gleason score 8). The line at the bottom left of the picture represents 100 µm. Panels B–D, effect of IL-4 upon the FAAH activity of human PC-3 prostate cancer cells (B); rat R3327 AT-1 prostate cancer cells (C) and mouse P19 teratocarcinoma cells (D). The cell lines were incubated for 24 h with 5 ng/ml of IL-4 and the FAAH activity in cell homogenates was determined using 2 µM [^3^H]anandamide as substrate. The enzyme activity in all cases was totally blocked by the selective FAAH inhibitor URB597. Data are means and s.e.m., n = 4–5; *p<0.05, **p<0.01, paired t-test. In Panels (E) and (F) PC-3 and LNCaP cells were incubated with IL-4 (5 ng/ml) for 24 h. Extraction of RNA, cDNA synthesis and PCR analysis were performed as described in the ‘Methods’ section. Panel E. Semi-quantitative PCR analysis of FAAH mRNA expression in PC-3 and LNCaP cells. The mRNA expression of FAAH was normalized against GAPDH. The semi-quantitative PCR analysis was performed using pooled samples (n = 6). Panel F. Quantitative real-time PCR analysis of FAAH mRNA expression, normalized against GAPDH. Quantitative PCR data are visualized as means (n = 6) and s.e.m, where the untreated control group mean is defined as 100%.

One explanation for the finding that the tumour epithelial FAAH-IR is higher than FAAH-IR in normal prostate epithelial tissue [12] is that a constituent of the tumour microenvironment affects its activity. The T_H_2 cytokine interleukin-4 (IL-4), which has been reported to regulate FAAH and CB_1_ receptors in lymphocytes and Jurkat cells [26]–[28], is involved the pathogenesis of several cancer types, including prostate cancer (see [29]–[35]). IL-4 receptors have been reported in both tumour epithelial cells and in cultured prostate cancer cell lines [29], [36]. We confirmed the presence of IL-4 IR in prostate cancer epithelial tissue (Fig. 2A), and found that incubation of both human androgen-insensitive PC-3 cells and rat Dunning R3327 AT-1 prostate cancer cells with IL-4 for 24 h produced a significant increase in FAAH activity of the cells (Fig. 2B and C). A similar result was seen with human prostate cancer LNCaP cells (data not shown). This effect of IL-4 is not unique to prostate cancer cells, since it was also seen mouse P19 teratocarcinoma cells (Fig. 2D).

In subsequent experiments, the effects of IL-4 treatment upon FAAH protein and mRNA were assessed. Treatment of PC-3 and LNCaP cells with IL-4 for 24 h did not regulate the mRNA expression of FAAH, as assessed by both semi-quantitative PCR and quantitative real-time PCR (Fig 2E, 2F). In an additional experiment, PC-3 cells were treated with IL-4 (5 ng/ml) for 3 h, to study possible early regulation of FAAH expression. Similar to the 24 h experiment, however, treatment of PC-3 cells with IL-4 for 3 h did not regulate the mRNA expression of FAAH (data not shown). In contrast to the situation for Jurkat cells and T-lymphocytes, the expression of CB_1_ in LNCaP cells was not regulated by IL-4: the relative expression of CB_1_/GAPDH (% of untreated controls, means ± s.e.m., n = 6) following 24 h of treatment with either vehicle or IL-4 was 100±33 and 109±20, respectively. The PC-3 cells used in our lab did not express detectable levels of CB_1_ mRNA (data not shown).

Preliminary Western blots analyses were also undertaken using lysates from PC3 cells treated for 24 h with either vehicle or 5 ng/ml IL-4. In addition to the expected molecular weight band, additional bands were seen suggesting that there may be protein aggregation and some breakdown in the lysates, post-translational protein modification by the cells, and/or that the assay protocol was not fully optimised, although three different dilutions of the primary antibody were used. However, none of the bands showed any obvious difference in intensity following IL-4 treatment, protein loading being confirmed with actin blots (data not shown).

### FAAH-IR in the tumour tissue: correlation with disease severity at diagnosis

In Table 2, the correlation coefficients for the tumour FAAH-IR and clinical variables determined at diagnosis are listed. For the complete dataset, there was a significant positive correlation of the epithelial, but not blood vessel, FAAH-IR with both the Gleason score and the % of specimen that contained tumour, whereas neither score correlated significantly with the incidence of metastases at diagnosis. A correlation was also seen for the epithelial, but not blood vessel FAAH-IR with the local stage number, the immunoreactive score for phosphorylated epidermal growth factor receptor (pEGFR, a marker for prostate cancer outcome [37], Table 1), and with cytoplasmic, but not nuclear HER2 (human EGF receptor 2, data not shown). No significant correlation was seen between either epithelial or blood vessel FAAH-IR and either the immunoreactive score for non-phosphorylated EGFR or the tumour vascular density (number of vWf-stained vessels; number of endoglin stained vessels) (data not shown). In the tumour stroma, the androgen receptor expression is inversely related to disease severity and outcome [38]. However, there was no significant correlation between the epithelial or blood vessel tumour FAAH-IR and the stroma androgen receptor IR in the 69 (epithelial) or 57 (blood vessel) cases which had been scored for both parameters (data not shown).

**Table 2:** Correlation of tumour FAAH-IR with clinical measures at diagnosis.

	Spearman's r [no of pairs in correlation] for tumour FAAH-IR in:					
Parameter:	Epithelial cells					blood vessels
CB_1_IR:	all	<2	2	2.025–2.25	≥2.3	all
Age	−0.07^NS^[388]	−0.28**[91]	0.04^NS^[114]	−0.15^NS^[83]	−0.06^NS^[82]	−0.05^NS^[360]
Gleason score	0.21***[388]	−0.12^NS^[91]	0.27**[114]	0.30**[83]	0.05^NS^[82]	−0.01^NS^[360]
Tumour stage	0.11*[382]	−0.31**[90]	0.21*[111]	0.18^NS^[82]	−0.004^NS^[81]	0.02^NS^[354]
% of the specimen that contained tumour	0.17**[388]	−0.23*[91]	0.24*[114]	0.20∧[83]	0.06^NS^[82]	0.006^NS^[360]
Incidence of metastases at diagnosis	0.10^NS^[303]	0.14^NS^[74]	0.10^NS^[90]	.004^NS^[58]	0.04^NS^[68]	0.02^NS^[281]
pEGFR	0.18**[293]	0.06^NS^[73]	0.10^NS^[91]	0.19^NS^[65]	0.16^NS^[49]	0.07^NS^[275]

***p<0.001, **p = 0.01, *p<0.05, ∧not significant (p<0.07) ^NS^not significant (p>0.08). The CB_1_IR values are taken from the original data of [14], and intervals chosen represent the data roughly divided into quartiles. pEGFR-IR values are taken from the original data of [37].

The correlation between the Gleason scores and the tumour epithelial FAAH-IR is illustrated in Fig. 1D, where the FAAH-IR is divided into four approximately equal (in sample size) ranges, from the lowest (“I”) to the highest (“IV”). As the FAAH-IR increases from I to IV, the number of cases with Gleason scores 4–5 decreases, and the number of cases with Gleason scores 8–10 increases. Thus, the FAAH-IR correlates with disease severity at diagnosis, despite the fact that the median FAAH-IR scores for each Gleason group are not that different (2 (n = 88), 2.075 (n = 102), 2.075 (n = 66) and 2.1 (n = 132) for Gleason scores of 4–5, 6, 7 and 8–10, respectively). Further analysis indicated that the significant correlation of the tumour epithelial FAAH-IR with the Gleason score was seen for mid-range CB_1_IR scores, but not for either low or high CB_1_IR scores (Table 2). The correlation with the tumour stage and the % of specimen that contained tumour was also dependent upon the CB_1_IR range used, being negative at low CB_1_IR scores, positive at the median CB_1_IR score (2), and not being seen at the high CB_1_IR scores. The correlation with pEGFR was not significant when the data was subdivided into the CB_1_IR quadrants (Table 1), suggesting that the correlation with FAAH-IR reflects a primary correlation of this measure with the CB_1_IR. Indeed, the correlation between tumour epithelial pEGFR and CB_1_IR was highly significant (Spearman's r value 0.30, n = 280, p<0.001).

### Tumour epithelial FAAH-IR is associated with disease outcome

**Figure 3:**
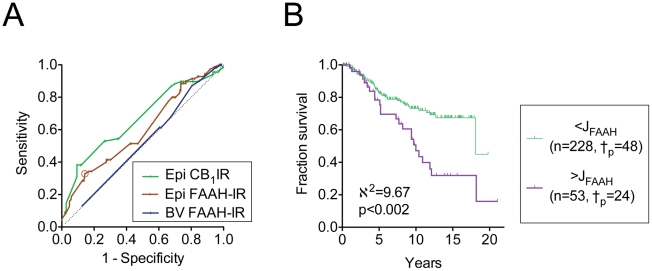
Association of FAAH-IR with prostate cancer outcome. Panel A. Receiver-operated characteristic (ROC) analyses for tumour epithelial (Epi) and blood vessel (BV) FAAH-IR. A 15 year cut-off was used for the patients who were not treated. For comparative purposes, the ROC curve for epithelial CB_1_ receptor IR (Epi CB_1_IR, from the original data of [14] is shown). The dotted line indicates the result if the parameter under study has no prognostic value (i.e. area under the ROC curve = 0.5). The AUC (95% confidence intervals in brackets) and significance levels were: Epi FAAH-IR: 0.598 (0.520–0.677), p<0.02); BV FAAH-IR: 0.516 (0.434–0.597), p>0.7; Epi CB_1_IR (from [14]), 0.665 (0.586–0.744), p<0.0001). The optimal cutoff (Youden value) for the Epi FAAH-IR (J_FAAH_) is shown as an open circle on its ROC curve. Panel B, Kaplan-Meier plot for the fraction survival of patients followed with active expectancy with tumour epithelial FAAH-IR scores of either below or above the J_FAAH_ value (2.354). The number of cases where death was due to prostate cancer are indicated as †_p_ in the figure. The hatches on the lines show censored data (i.e. cases other than death due to prostate cancer). The 15-year probabilities of event-free survival for FAAH-IR below and above the J_FAAH_ value were 67±4% and 32±9%, respectively.

During the period when the specimens were collected, the standard treatment for prostate cancer was watchful waiting until the appearance of metastases. Thus, a substantial proportion of the samples represent essentially untreated patients, which allows for the evaluation of the association of biological parameters measured at diagnosis upon the outcome of the disease. Receiver operating characteristic (ROC) analyses were undertaken on the data subset for the untreated patients (for an explanation of the methodology to the reader not well versed in this statistical approach, see the statistics section of Methods). For the tumour epithelial FAAH-IR, the area under the ROC curve using a 15 year cutoff (259 cases) was significantly above 0.5, and the optimal cutoff point (Youden Index, here termed J_FAAH_) for the epithelial FAAH-IR was determined to be at a score of >2.354 (Fig. 3A). The area under the ROC curve for the blood vessel FAAH-IR was not significantly different from 0.5 (Fig. 3A). Using this J_FAAH_, Kaplan-Meier analyses demonstrated a significant contribution of tumour epithelial FAAH-IR upon the disease outcome (Fig. 3B). The median survival (i.e. the time at which fractional survival is 50%) was 18.1 vs. 10 years for the patients with an FAAH-IR below vs. above J_FAAH_. The hazard ratio for the two curves (Mantel-Haenszel method) was 2.574 (95% confidence interval 1.418–4.671).

COX proportional-hazards regression analyses were undertaken using different cutoff values for CB_1_IR to assess the extent to which FAAH-IR provided additional prognostic information to that given by the CB_1_IR score. Using a cutoff CB_1_IR value of ≥2 (the cutoff used in [14]), a significant contribution by FAAH-IR was seen in the multivariate analysis (Table 3). A similar significance was seen using a CB_1_IR cutoff value of ≥2.1 (FAAH Exp(B) = 1.969, p<0.01). However, at a CB_1_IR cutoff of ≥2.3, the significant contribution of FAAH was lower (Table 1), and at a CB_1_IR cutoff of ≥2.5, the contribution of FAAH was lost (FAAH Exp(B) = 1.623, p = 0.073). The contribution by CB_1_IR was significant at all cut-offs. These data indicate that the additional information provided by FAAH-IR decreases as the CB_1_IR cut-off used increases. In contrast to CB_1_IR [14], Cox proportional-hazards regression analyses indicated that FAAH-IR did not provide additive prognostic information to Gleason scores (data not shown).

**Table 3:** Cox proportional-hazards regression analyses for tumour epithelial FAAH-IR and CB_1_IR scores.

Variable	Categorical value	n	Exp(B)^1^	95% CI	p
*Univariate analysis*					
FAAH-IR	<J_FAAH_ = 0	228			
	>J_FAAH_ = 1	53	2.151	1.311–3.528	0.005
CB_1_IR	<2 = 0	77	3.244	1.609–6.543	<0.001
	≥2 = 1	192			
CB_1_IR	<2.3 = 0	225	4.402	2.688–7.210	<0.001
	≥2.3 = 1	44			
*Multivariate analysis*					
FAAH-IR	<J_FAAH_ = 0	217			
	>J_FAAH_ = 1	50	2.189	1.318–3.635	0.002
CB_1_IR	<2 = 0	77			
	≥2 = 1	190	3.354	1.660–6.774	<0.001
*Multivariate analysis*					
FAAH-IR	<J_FAAH_ = 0	217			
	>J_FAAH_ = 1	50	1.690	1.007–2.839	0.047
CB_1_IR	<2.3 = 0	223			
	≥2.3 = 1	44	3.949	2.385–6.540	<0.001

1 For those readers not familiar with the output of a Cox proportional-hazards regression analysis, exp(B) refers to odds ratio, i.e. the change in odds as a result of an increase in the “unit” of the predictive variable under study. For example, FAAH-IR<Y_FAAH_ and >Y_FAAH_ are given categorical “units” of 0 and 1, respectively (see [39]). The J_FAAH_ value is 2. 354.

## Discussion

The main findings of the present study are that FAAH-IR is associated with prostate cancer disease severity and outcome, that FAAH-IR and CB_1_IR give additive prognostic information at mid-range, but not high CB_1_IR, and that the correlation of FAAH-IR and CB_1_IR in the tumour, but not in the non-malignant, tissue is consistent with a local dysregulation due to a component of the tumour microenvironment. These findings raise four questions:

### 1. Are the FAAH-IR seen in the tumour tissue consistent with the literature

To our knowledge, the only study of FAAH in human prostate cancer tissue samples [12] used a different FAAH antibody and a commercially available tissue microarray. These authors found a low incidence of FAAH-IR in normal tissue (1 of 8 cores) whereas a FAAH expression was seen in 109 of 157 cores for cases of prostate cancer. In benign prostate hyperplasia the basal cells expressed FAAH, whereas the luminal cells showed weak FAAH-IR [12], a finding entirely consistent with our data with the non-malignant tissue samples. Those authors found that although the mean FAAH-IR was higher in the tumour samples than in the normal tissue, there was no obvious relation to the Gleason scores (grouped as 2–4, 5–6 and 7–10) [12]. Our data using a much larger sample indicates that the medians are similar for the different Gleason groups, but that there is a significant correlation between the tumour epithelial FAAH-IR and the Gleason score, and that there is a higher proportion of cases with a high Gleason score (8–10) in cores with a high tumour epithelial FAAH-IR than those with a low FAAH-IR. In prostate cancer, the predominant epithelial cell type stains for markers of luminal cells [40], and our finding that the median FAAH-IR in the epithelial tumour cells was higher than in the luminal cells in the non-malignant cores would be consistent with the notion of an up-regulation of FAAH in the tumour tissue.

To our knowledge, the only study investigating FAAH as a potential prognostic factor for disease outcome is in pancreatic cancer [41]. These authors found that in a cohort of 29 patients, cases with a low FAAH-IR showed a poorer prognosis than cases with a high FAAH-IR. The authors also investigated mRNA using quantitative PCA in a separate and larger cohort (n = 53) and found that FAAH provided no prognostic information [41]. The authors pointed out that there were a low number of cancer cells in the tissue preparations and this, together with the low cohort sizes, may explain the difference between the mRNA and the IR data. An additional consideration is the choice of cutoff. In the present study, the ROC curve for FAAH had an early (but small) peak, then returned to the line of no diagnostic value before peaking at the Youden value (shown circled in Fig. 3A. A simple median split of our data (i.e. essentially as undertaken in [41] as far as can be ascertained from their Kaplan-Meier curves) would give cutoffs here of ≤2 and >2, which is in the region of the ROC curve where the FAAH-IR lacks prognostic value, and indeed the Kaplan-Meier plots for these two groups are superimposible, with 15 year disease-specific survival rates of 62±6% and 56±6% for the cases with FAAH-IR scores ≤2 (n = 143) and >2 (n = 138) (data not shown). This is in contrast to the situation for the CB_1_IR, where the ROC curve is more robust (see Fig. 3A) and the choice of cutoff is less critical.

### 2. Is measurement of FAAH in prostate tumour tissue obtained at diagnosis a potential prognostic marker

The scoring system used in the present study, whereby both intensity and distribution are measured, is useful for evaluating the role of FAAH in the pathogenesis of prostate cancer, which was the aim of the present study, but may be too complex for routine clinical diagnosis, where a simple intensity score would be preferable. Even with this caveat, the answer to the question is “no”, since a) the area under the ROC curve, albeit significantly higher than 0.5 (the score for no prognostic value), is modest, and b) the FAAH-IR does not provide significant additional information to that provided by the Gleason scores. A consequence of this modest ROC value is that the test-retest reliability of the measure (as assessed by investigating the contribution of the FAAH upon disease-specific survival of random splits of the data set) is not good (data not shown). This is in contrast to the situation for CB_1_IR, which has a higher area under the ROC curve (0.67), which shows a robust test-retest reliability, and which provides significant additional prognostic information to that to that provided by the Gleason scores [14].

### 3. How do FAAH and CB_1_IR contribute to disease severity and outcome

The data presented here indicate that the degree to which FAAH is associated with disease severity and outcome are dependent upon the prevailing CB_1_IR. Thus, for example, for the 114 samples with a CB_1_IR value of 2, there is a clear correlation of FAAH with the Gleason score, whereas this is not seen for the samples with a CB_1_IR≥2.3 or for CB_1_IR<2. In the Cox proportional-hazards regression analyses, FAAH-IR provided significant additional information to that provided by CB_1_IR at CB_1_IR cutoffs of ≥2 and ≥2.1, but not at ≥2.5. It is thus worth considering the role of FAAH at “mid-range” and “high” levels of CB_1_ expression.

There are elegant studies for prostate cancer cells showing that endocannabinoid levels control invasive behaviour in an *in vitro* model. Thus, the metabolically stable anandamide analogue methananadmide and the synthetic cannabinoid WIN55,212-2 reduce the invasivity of androgen-resistant PC-3 and DU-145 (but not the androgen-sensitive LNCaP) cells, whilst depletion of 2-arachidonoylglycerol or addition of the CB_1_ receptor antagonist rimonabant increases the invasivitiy of the androgen-resistant cells [8]. PC-3 cells have relatively low levels of FAAH, and transfection of the cells with FAAH increases both their migration and invasivity *in vitro* [12]. Although the step from *in vitro* models to man is large, these data do provide a framework for understanding how a high FAAH-IR, by reducing the local endocanninoid levels, is associated with disease outcome in cases with a mid-range expression of CB_1_IR – in the cases with a low CB_1_IR, the target for endocannabinoid action is absent. In this respect, it is worth pointing out that although 2-AG is usually regarded as a substrate for monoacylglycerol lipase, FAAH is capable of hydrolysing this substrate [42], and in some tissues and cells, inhibition of FAAH results in an increased level of both AEA and 2-AG [43]–[45]. The relative importance of FAAH and MGL in the metabolism of 2-AG has been investigated in prostate cancer cells. In intact PC-3 cells, exogenous 2-AG is hydrolysed by an OTFP (3-octylthio-1,1,1-trifluoropropan-2-one)-sensitive process to arachidonic acid, which is then metabolised by 12-lipoxygenase to form 12-hydroxyeicosatetraenoic acid [46]. In our hands, the hydrolysis of 2-AG by intact PC-3 cells is only marginally inhibited by the selective FAAH inhibitor URB597, and in the case of R3327 AT-1 cells, no inhibition is seen [47]. However, transfection of PC-3 cells with FAAH does increase their rate of 2-AG hydrolysis in addition to the effects on AEA hydrolysis [12].

The above discussion assumes that the CB_1_ receptors are the target for the local endocannabinoids, and certainly the *in vitro* invasivity data using prostate cancer cell lines would support this contention [8]. A loss of CB_1_ receptors would also, according to this assumption, be deleterious, and this has been reported in colorectal cancer [48]. The finding that in hepatocellular carcinoma, individuals with a low expression of CB_1_ and CB_2_ receptors have a poorer prognosis than individuals with a high expression of these receptors [49] would suggest that a similar mechanism may also be operative in this cancer form. However, this does not exclude other potential targets of endocannabinoid, or endocannabinoid metabolite, action. TRPV1 receptors are expressed in human prostate tissue [50], [51], so in theory some of the protective properties of AEA may involve activation of these receptors, given that in other cancer cell types, AEA can produce apoptosis via such a mechanism [52]. To our knowledge, this possibility has not been investigated in prostate cancer cells, although the effects of [R]-methanandamide, the metabolically stable analogue of anandamide, upon the viability of PC-3 cells are mediated by CB_2_ rather than TRPV1 receptors [53]. In addition, it has been proposed that free fatty acids produced from the corresponding 2-acylglycerols (including 2-arachidonoylglycerol) by the action of monoacylglycerol lipase may be involved in the pathogenesis of several tumour types [54], and it can be argued that FAAH may also play this role in the generation of free fatty acids from the corresponding *N*-acylethanolamines (including anandamide).

At variance with the above discussion is the finding that the prognostic information supplied by FAAH is lost at a high CB_1_IR, and indeed, in both prostate and pancreatic cancers, a high rather than a low CB_1_ expression is associated with a poorer outcome [14], [41]. In the pancreatic cancer, this finding was confirmed when mRNA levels were measured in a separate cohort [41]. However, a recent article in this Journal has shed light on one possible mechanism for this variation [55]. These authors constructed astrocytoma subclones expressing different levels of CB receptors, and found that the signalling pathways involved upon activation of the receptors were dependent upon the receptor expression level. At low levels of receptor expression, the receptors coupled to Erk1/2 and cannabinoids produced apoptosis. At high levels of receptor expression, this pathway was still operative, but the receptors also coupled to Akt, and cannabinoids only produced apoptosis when this pathway was inhibited [55]. If this is a generalised phenomenon, then a case can be made that in prostate cancer, patients with low CB_1_IR are sensitive to apoptosis produced by local endocannabinoids (which will be regulated by FAAH and monoacylglycerol lipase), whilst cases with high CB_1_IR will be insensitive to the local endocannabinoid levels. Needless to say, the step from astrocytoma cells *in vitro* to prostate cancer is large, and factors such as changes in receptor reserve (seen in normal tissues with a high CB_1_ expression, such as the hippocampus [56]) may also be of importance. Nonetheless, the receptor-dependent signalling pathways described in [55] would explain why the influence of a high FAAH-IR is greater at low CB_1_IR than at high CB_1_IR scores.

A second factor that may be involved in the poor prognosis seen for patients with a high CB_1_IR relates to the activation of the pEGFR pathway. The present study shows that in the tumour epithelia, FAAH-IR is correlated with the pEGFR-IR, but that this is secondary to a correlation between CB_1_IR and pEGFR-IR. EGFR is an important signalling protein, and aberrant signalling has been implicated in the pathogenesis of several cancer types, including prostate cancer (see [37], [57]). In human U373-MG glioblastoma cells, cannabinoids induce a cell proliferation that is dependent upon the EGFR signalling pathway [58] and in C6 rat glioma cells, expression of the EGFR ligand amphiregulin is an important factor controlling their resistance to the apoptosis produced by cannabinoids [59]. The anti-proliferative effect of anandamide in prostate cancer cells has also been associated with a down-regulation of EGFR expression [7].

### 4. Why is FAAH-IR expression higher in tumour tissue than in the normal prostate

Our correlation data with FAAH and CB_1_ provide useful data in this respect. In theory, there should be no correlation between two completely independent variables, unless both variables are both affected in the same manner by a third variable. FAAH and CB_1_ receptors are located on different chromosomes [60], [61] and do not appear initially to have co-evolved [62], so there is no compelling reason why their expression should be correlated in normal tissue. Indeed, in the non-malignant tissue (which is admittedly far from normal), there was no correlation at all between the CB_1_IR and the FAAH-IR. This is important in itself, since it would suggest that the two measures are independent, and that a high CB_1_IR is not a homeostatic consequence of a low endocannabinoid signalling secondary to a high FAAH expression. However, a correlation was seen in the tumour tissue. It is well known that the tumour microenvironment plays an important role in the pathogenesis of prostate cancer [63], and it can be hypothesised that a component of the tumour microenvironment regulates in the same manner the activity of both CB_1_ receptors and FAAH. The observed expression levels will thereby show a degree of co-variance depending upon the levels of the micro-environmental regulator, and hence a correlation will be seen.

Although there are a number of potential regulatory sites on the FAAH promoter [64], few studies have investigated the regulation of this enzyme in cell systems. To our knowledge the only molecules known to regulate FAAH activity are the T_H_1 cytokines IL-12 and IFNγ (which decrease FAAH activity in lymphocytes), progesterone, leptin, the T_H_2 cytokines IL-4 and IL-10 (which increase FAAH activity and FAAH protein content in lymphocytes), and follicle stimulating hormone (which increases FAAH activity in Sertoli cells) [26], [65], [66]. None of these molecules have been investigated in prostate cancer cells with respect to FAAH. IL-4, which is found in the stroma of tumour regions of the prostate, upregulates anti-apoptotic proteins in prostate cancer cells [29]. Importantly for the co-variance argument raised above, IL-4 also increases the expression of CB_1_ receptors in lymphocytes [27], [28] so we elected to focus on that molecule as a “test of concept” that a component of the tumour microenvironment can influence FAAH activity. We could confirm that tumour epithelial cells express IL-4 receptors, in line with previous studies using both tumour tissue and cultured cells [29], [36], and could demonstrate that IL-4 increases FAAH activity in a PC-3 and R3327 AT1 prostate cancer cells as well as in P19 teratocarcinoma cells, indicating it to be a rather general effect of this cytokine. However, and at variance with the lymphocyte study of [26], we did not observe any obvious increase in FAAH protein levels following IL-4 treatment, although as a caveat it should be pointed out that the Western blot protocol we used was not fully optimised. There were also no changes in mRNA levels for FAAH in either PC3 or LNCaP cells following incubation with IL-4 for either 3 or 24 h. One possible explanation for these results is that IL-4 treatment results in a change either in the membrane conformation or in the cellular disposition of the FAAH in the prostate cancer cells, so that more is available to the substrate without the need for *de novo* synthesis, but such an explanation requires further study.

In conclusion, the present study has demonstrated that tumour epithelial FAAH-IR is associated with prostate cancer severity and outcome. A case is made that the FAAH-IR is regulated by the tumour microenvironment, and a potential candidate molecule, IL-4, has been investigated *in vitro*, although contributions by other components of the tumour environment should certainly be considered. The present data motivate studies designed to determine how IL-4 regulates FAAH in prostate cancer cells compared to lymphocytes, whether stromal IL-4 immunoreactivity (and/or expression of IL-4 receptors in the tumour epithelia) correlates with tumour epithelial FAAH-IR in prostate cancer tissue, and which other components of the tumour microenvironment are potential regulators of the endocannabinoid system.
